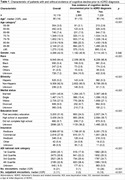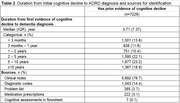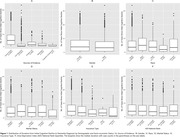# Tracking the timeline: from initial documentation of cognitive decline to dementia diagnosis in the electronic health records

**DOI:** 10.1002/alz.095745

**Published:** 2025-01-09

**Authors:** Liqin Wang, Richard Yang, David W Bates, Li Zhou, Gad A Marshall

**Affiliations:** ^1^ Brigham and Women’s Hospital, Harvard Medical School, Boston, MA USA; ^2^ Massachusetts General Hospital, Brigham and Women’s Hospital, Harvard Medical School, Boston, MA USA

## Abstract

**Background:**

Recent breakthroughs in the treatment of Alzheimer’s Disease underscore the critical needs for early detection. In clinical settings, the diagnosis of Alzheimer’s disease and related dementias (ADRD) is often delayed or overlooked, potentially missing critical opportunities for timely care and treatment. Cognitive symptoms can appear many years before the stage of dementia, potentially serving as a trigger for thorough cognitive assessments, which could prompt an early diagnosis of cognitive decline. However, it remains unclear how this information is presented to clinicians and documented in patient charts, and how early these symptoms appear before an ADRD diagnosis.

**Method:**

This retrospective cohort study utilized electronic health records (EHRs) from Mass General Brigham (MGB) to examine primary care patients aged 55 and older who received an initial ADRD diagnosis at MGB between 2018 and 2019. We analyzed EHR data to identify evidence of cognitive decline preceding the ADRD diagnosis. This analysis involved searching for cognition‐related keywords and diagnostic codes, and screening clinical notes using a validated deep learning model for cognitive symptoms. We assessed the duration from initial cognitive symptoms to ADRD diagnosis and examined demographic and socio‐economic factors for their correlation with documentation of cognitive decline.

**Result:**

The study included 10,119 ADRD patients (median age of 80, 42.5% male). Of these, 2,890 (28.6%) had no prior documented cognitive decline. Patients without prior documentation were slightly older at diagnosis (median age: 81 years), more likely to have unspecified education background (39.2% vs. 20.3%), and came from more deprived areas. Among patients with prior cognitive decline documented, the average time to ADRD diagnosis was 3.7 years. Patients with initial cognitive evidence from clinical notes (78.7%, n = 5,692) experienced earlier documentation of cognitive symptoms compared to those identified through other sources. In contrast, those without documented marital status, with Medicaid insurance, and residing in highly deprived neighborhoods experienced delayed documentation of cognitive symptoms prior to ADRD diagnosis.

**Conclusion:**

Our analysis reveals significant disparity in documenting cognitive decline among socio‐economically disadvantaged groups. This underscores the need for targeted outreach and intervention, and reinforces the role of comprehensive documentation in improving patient outcomes by facilitating earlier diagnosis and treatment.